# Dorsal penile nerve block versus eutectic mixture of local anesthetics cream for pain relief in infants during circumcision: A meta-analysis

**DOI:** 10.1371/journal.pone.0203439

**Published:** 2018-09-06

**Authors:** Jiamin Wang, Shankun Zhao, Lianmin Luo, Yangzhou Liu, Zhiguo Zhu, Ermao Li, ZhiGang Zhao

**Affiliations:** 1 Department of Urology & Andrology, Minimally Invasive Surgery Center, Guangdong Provincial Key Laboratory of Urology, The First Affiliated Hospital of GuangZhou Medical University, Guangzhou, Guangdong, China; 2 Research Lab for Clinical & Translational Medicine, Medical School, University of South China; Hengyang, China; Weill Cornell Medical College, UNITED STATES

## Abstract

**Objective:**

To compare dorsal penile nerve block (DPNB) and eutectic mixture of local anesthetics (EMLA) cream for pain relief in infants during circumcision.

**Methods:**

We systematically searched Medline via PubMed, Embase, CNKI and the Cochrane Library Center Register to identify randomized controlled trials up to March 2018. Effect estimates were performed in random effect models. Mean neonate infant pain scale (NIPS) scores, incidence of hematoma, edema and erythema, mean heart rate were conducted to assessed the effect of analgesia. We found that the EMLA had significantly higher pain scores compared to DPNB (SMD = 3.72, 95% CI 1.27–6.17, *P* = 0.003). In DPNB group, the incidence of hematoma was significantly higher than EMLA group, OR = 0.03, 95% CI 0.00–0.24, *P* = 0.001. The analysis did not show any significant differences in mean heart rate and the risk of edema and erythema between EMLA and DPNB group (SMD = 21.71, 95% CI = -0.88–44.30, *P* = 0.06 & OR = 0.40, 95% CI 0.15–1.07, *P* = 0.07 & OR = 7.33, 95% CI 0.84–64.07, *P* = 0.07).

**Conclusion:**

Based on the pooled results from the included studies, we found that DPNB was significantly more effective in pain relief as indicated by mean NIPS score than EMLA in infants during circumcision. However, use of DPNB significantly increased the risk of hematoma.

## Introduction

Male circumcision is a common procedure that has been performed for thousands of years for phimosis [1]. Inadequate pain relief in neonates in the perioperative period may have long-term physiological consequences, such as response to future painful stimuli and altered sensory processing [2,3]. The basic requirements for managing pain following pediatric circumcision are patient safety, reliability, rapid recovery, a low risk of adverse effects and ease of administration of a painless technique.

Commonly used methods of analgesia are distal, ring, or dorsal penile nerve block (DPNB), and eutectic mixture of local anesthetics (EMLA) cream [4]. The most widely studied and acceptable method for pain relief is DPNB [5]. However, it requires some experience to administer and has been association with analgesia at foreskin separation. This technique is associated with a 4%-8% incidence of failure and 5% incidence of hematoma [6].

EMLA cream is currently being used in the pediatric population for invasive procedures such as lumbar puncture, intravenous insertion, and central line insertion [7]. In recent years, urologists have tried to use it as a local anesthesia for circumcision. The usual dose for children and adults is 1-2g applied under an occlusive dressing for approximately 1 hour prior to the procedure. Benini et al [7] evaluated the efficacy of EMLA cream as an anesthetic agent in 21 newborns undergoing circumcision. Newborns in Benini’s study who had an application of EMLA 45–60 minutes prior to circumcision showed less increase in heart rate, less decrease in oxygen saturation, fewer facial actions, and less crying associated with pain. Although EMLA cream offers another anesthesia technique for minor surgical procedures on the penis, its effectiveness for such procedures has not been clearly evaluated. Clinicians have been unable to reach a consensus regarding analgesia during circumcision.

Therefore, we conducted this systematic review and meta-analysis to summarize the randomized controlled trials with the purpose of assessing the anesthetic effect of DPNB and EMLA cream of pain relief for circumcision.

## Methods

### Search strategy

**Fig 1:**
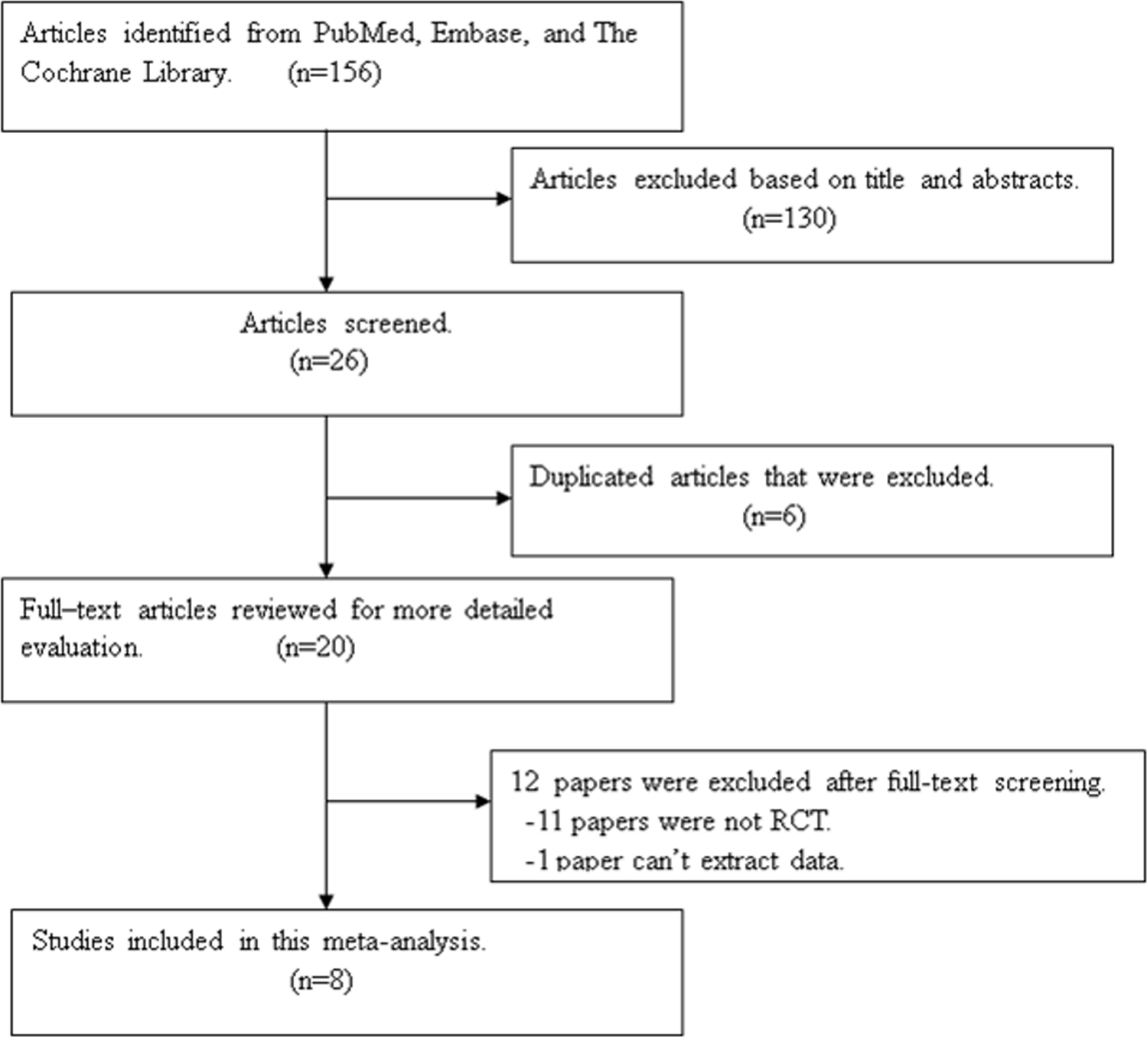
Flow chart of study selection.

We searched widely used databases (i.e. Embase, Cochrane library, CNKI and Medline via PubMed) for the published prospective randomized controlled trials from the beginning of indexing for each database through March 2018. English and Chinese were imposed in the search strategy. The following subject headings and keywords were used for each electronic databases: “male circumcision”, “dorsal penile nerve block” and “eutectic mixture of local anesthetics cream”. The full electronic search strategy in PubMed that were (((2-(Diethylamino)-N-2,6-Dimethylphenyl)Acetamide)OR((((((((((((((((("Lidocaine"[Mesh]) OR Lidocaine Monohydrochloride, Monohydrate) OR Dalcaine) OR Xylocitin) OR Xylocaine) OR Xylesthesin) OR Octocaine) OR Lidocaine Sulfate (1:1)) OR Xyloneural) OR Lidocaine Monoacetate) OR Lidocaine Monohydrochloride) OR Lidocaine Hydrochloride) OR Lidocaine Hydrocarbonate) OR Lidocaine Carbonate) OR Lidocaine Carbonate (2:1)) OR Lignocaine) OR 2-2EtN-2MePhAcN))) AND (((("Circumcision, Male"[Mesh]) OR Male Circumcision) OR Circumcisions, Male) OR Male Circumcisions). We searched for additional randomized controlled trials by examining the reference lists of the articles and published reviews. The diagram represents the flow of identification and inclusion of trials, as recommended by the Preferred Reporting Items for Systematic reviews and Meta-Analyses (PRISMA) statement [8] (Fig 1).

### Study selection

To draw a reliable conclusion, randomized controlled trials that compared the anesthetic effect of EMLA and DPNB in circumcision were included only. On the basis of patient, intervention, comparison, outcome and study design (PICOS), the question that guided this systematic-review was: Does EMLA has a better anesthesia effect as compare to DPNB in circumcision? The PICOS evidence base used consisted of the following combinations: older children and neonates (P); DPNB or EMLA (I); comparison of the DPNB and EMLA using RCT (C); the neonate infant pain scale (NIPS) scores, hematoma, edema and erythema (O); we only accepted randomized controlled trials (S). Studies were not limited by years of publication or location. Titles and abstracts of identified publications were screened by trained reviewers. Publications were excluded if they: were duplicates; were not in English or Chinese; were observational studies or case series. After full-text screening, studies of combination anesthesia of EMLA or DPNB were excluded. For each potential included study, two investigators independently carried out the selection evaluation, data abstraction, and quality assessment. Disagreements were resolved by discussion or in consultation with a third author when two investigates independently selected studies for inclusion in this study.

### Data extraction

All these information were recorded in a standardized form and the following data were sought from each study: year of publication, author’s first name, and study population, exact number of participants both in EMLA and DPNB groups, intervention measure, evaluation index and side effects as well (Table 1).

**Table 1:** Summary of studies of the comparison of the effect of pain relief of dorsal penile nerve block and lidocaine cream in circumcision.

study	year	study population	No. in E group	No. in D group		Intervention measure	Evaluation Index	Side effects
Butler-O'Hara, M et al	1998	24-60h	21	23	E: 0.5g EMLA D: 1% lidocaine (0.3–0.5ml)		HR, NIPS	erythema, hematoma, edema
Choi, W. Y et al	2003	2–12 years	30	30	E: 2~4g EMLA cream D: 0.5% plain bupivacaine (0.2 ml/kg)			erythema, hematoma, edema
Garry, D. J et al	2006	36h	6	6	E: 5%EMLA D: 1% lignocaine (0.3–0.5ml)		NIPS	None
Howard, C. R et al	1999	≥ 24h	31	31	E: 1g EMLA D: 1% lidocaine (0.8ml)		HR	None
Lee J J et al	1992	2~10 years	10	9	E: 2g EMLA D: 0.5% bupivacaine (1 mg/kg)			None
Lehr, V. T et al	2005	≤ 1 week old	17	18	E: 2g EMLA D: 1% lidocaine (0.4~0.6ml)			erythema, hematoma, edema
Salgado, F. M et al	2011	2–13 years	20	21	E: 5% lidocaine and 5% prilocaine (1.0g/10cm2) D: 0.5% bupivacaine (1 mg/kg)			erythema, hematoma, edema
Peng et al	2015	8 years	599	599	E: 10% EMLA (1.5g/10cm2) D: 2% lidocaine (1mg/kg)			None

E = EMLA group; D = DPNB group.

### Quality assessment

We assessed the safety and efficacy by merging side effects and evaluation index including heart rate (HR), neonatal NIPS. The quality of included studies were assessed using Cochrane’s risk of bias assessment tool. In addition, sensitivity analysis was performed with the method of calculating the unadjusted pooled OR by repeating the overall analysis after omitting each study in turn.

### Statistical analyses

Data were analyzed using the Review Manager 5.1.2 statistical package (Cochrane Collaboration Software) [9], and the clinical outcomes were reported as odds ratio (OR). The corresponding 95% confidence interval (95% CI) was calculated, considering P values less than 5% (*P*<0.05). A statistic for measuring heterogeneity was calculated through *I*^2^ method (25–50% was considered low-level heterogeneity, 50–75% moderate-level heterogeneity and >75% high-level heterogeneity). We carried out an additional analysis using the random-effects model described by e.g. DerSimonian et al [10], to see if there was statistical heterogeneity found in the meta-analysis. We executed the funnel plot test described by e.g. Egger et al [11] to determine the possibility of any publication bias. For all analyses, a forest plot was generated to display results. Also, we have deposited our laboratory protocols of the meta-analysis in protocols.io, please access at dx.doi.org/10.17504/protocols.io.vd9e6.

## Result

In the first search, 156 references were identified and screened. 130 studies were excluded as unmatched titles and abstracts. After full text review, 6 duplicated studies were excluded and 12 papers were excluded because they were not randomized controlled trials. The lasted 8 studies were what we needed [12–19].

A total of 8 studies involving 1571 patients were included in this review. The baseline characteristics of the included studies are shown in Table 1. Included studies were published between 1992 and 2015.

**Fig 2:**
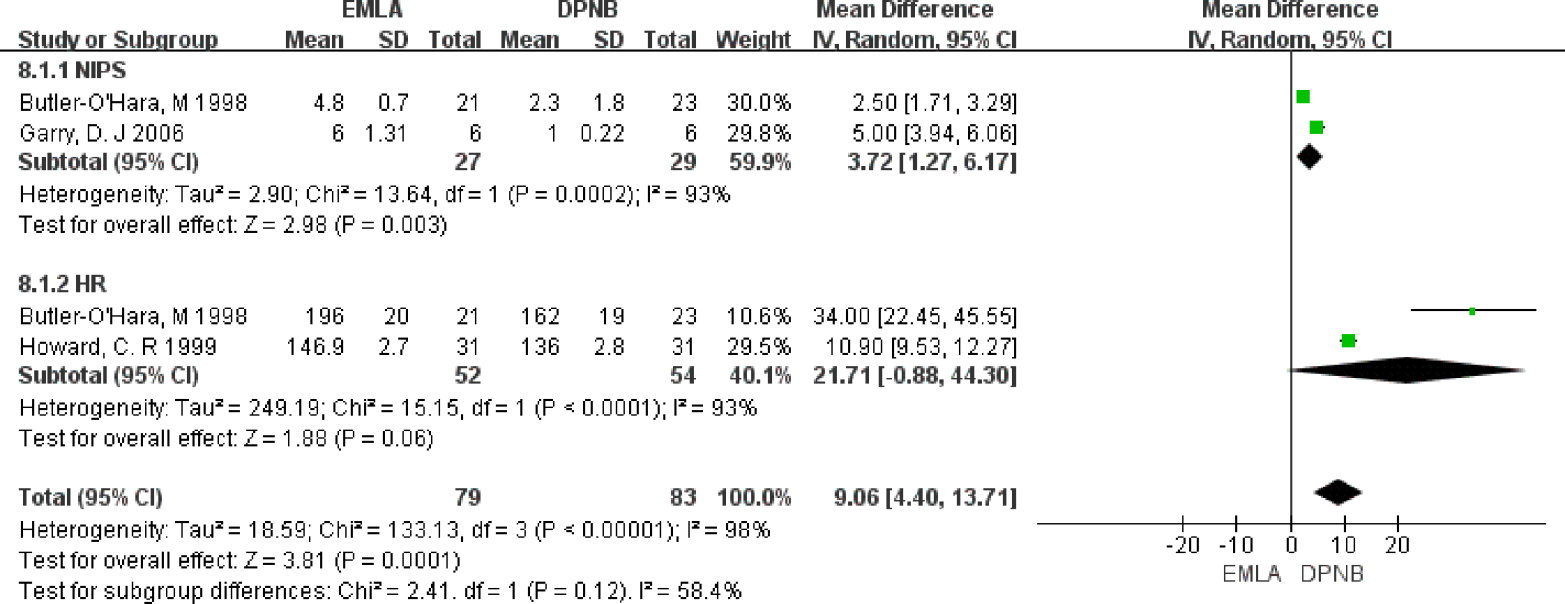
Comparison of NIPS and mean HR.

**Fig 3:**
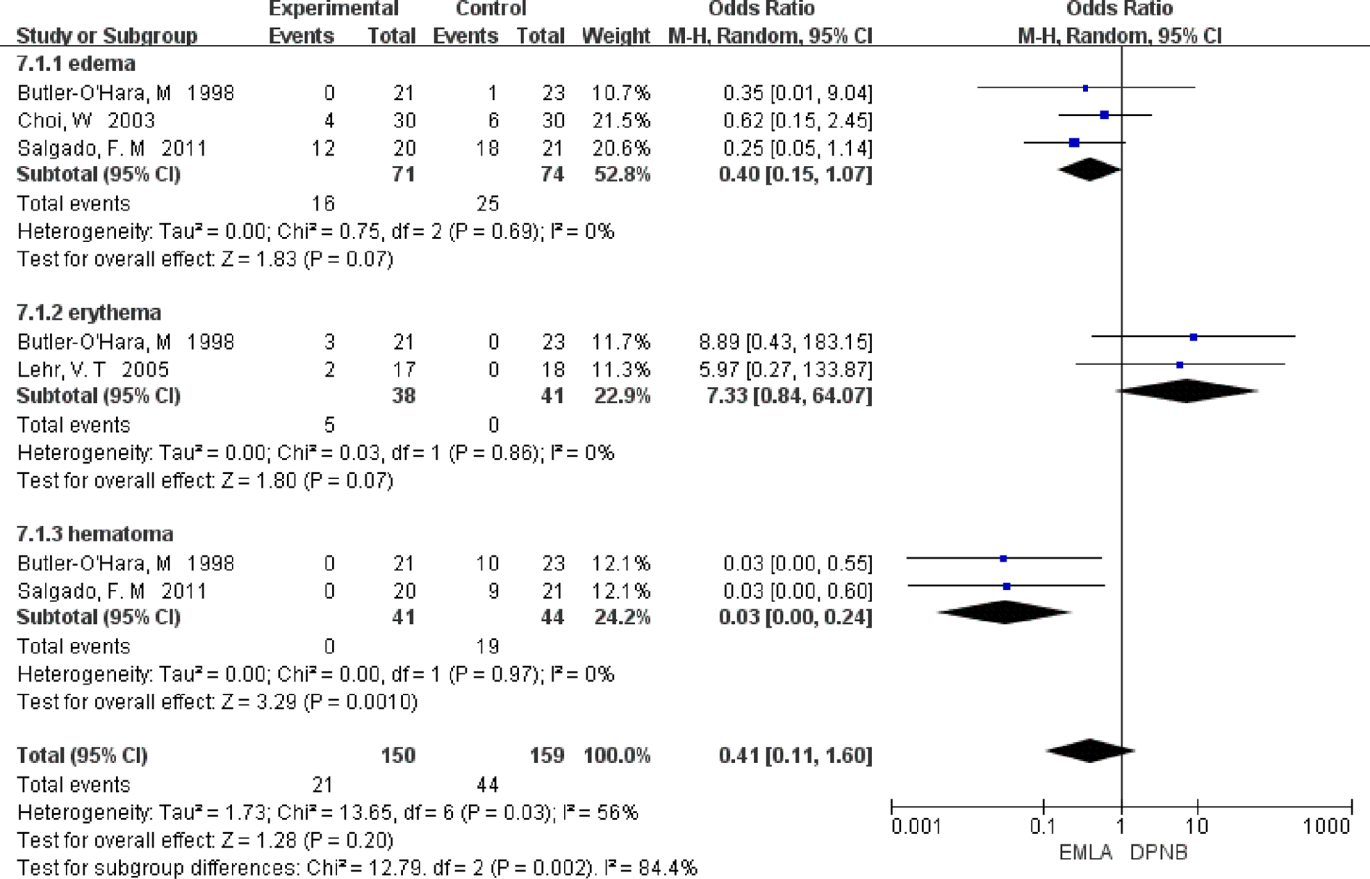
Comparison of edema, erythema, hematoma and secondary anesthesia.

We found that use of EMLA was associated with significantly higher NIPS scores compared to DPNB, which is standard mean difference (SMD) = 3.72, 95% CI 1.27–6.17, *P* = 0.003 (Fig 2). As to the mean HR during intra-operation, we did not find any significant difference between the EMLA and DPNB groups (SMD = 21.71, 95% CI = -0.88–44.30, *P* = 0.06) (Fig 3).

In DPNB group, the incidence of hematoma was significantly higher than EMLA group, OR = 0.03, 95% CI 0.00–0.24, *P* = 0.001. The analysis did not show any significantly differences of the risk of edema and erythema between EMLA and DPNB groups, correspondingly, that was OR = 0.40, 95% CI 0.15–1.07, *P* = 0.07, and OR = 7.33, 95% CI 0.84–64.07, *P* = 0.07 (Fig 3).

**Fig 4:**
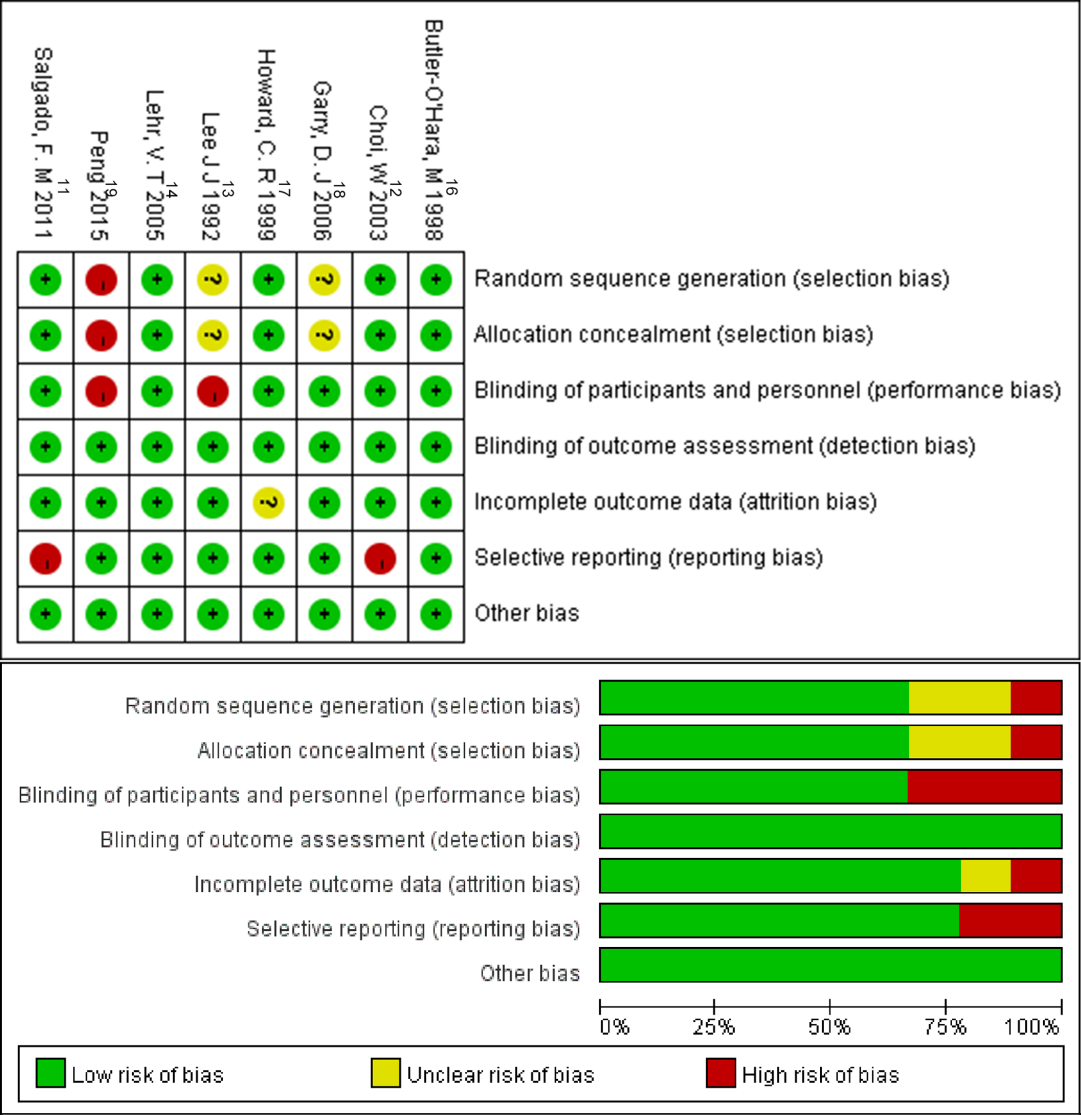
Risk of bias graph and risk of bias summary.

The Cochrane collaboration’s tool was used for assessing the quality of the study and the risk of bias. Lower risk in each bias were ranged from 62.5% to 100% (Fig 4).

Sensitivity analysis was utilized to detect the influence of each study on the pooled OR by repeating the meta-analysis, while omitting 1 single study each time. All studies in the comparison of edema were omitted each study in turn when repeating the overall analysis, it ranged from 0.27 [0.07, 1.05], *P =* 0.06 to 2.88 [0.11, 78.73], *P* = 0.53. There was no individual study significantly affected the pooled OR (Table 2).

**Table 2:** Sensitivity analysis after each study was excluded by turns.

comparison of edema	OR (95% CI) for remainders	Heterogeneity	
		I^2^ (%)	P
Butler-O'Hara, M et al	0.41 [0.15, 1.13]	0	0.09
Choi, W et al	0.27 [0.07, 1.05]	0	0.06
Salgado, F. M et al	0.56 [0.16, 2.01]	0	0.38

CI = confidence interval; OR = odds risk.

## Discussion

To the best of our knowledge, this is the first meta-analysis which compares the effect of pain relief between EMLA and DPNB in infants during circumcision. Based on the pooled results from the included studies, we found that DPNB was significantly more effective in pain relief as indicated by mean NIPS score than EMLA. At the same time, the DPNB have significantly improved the risk of hematoma. The lidocaine can be injected into circulation, hematomas can form at the site of injection, and the procedure is painful. However, our analysis have not sufficient behavioral data assessing the degree of pain associated with the DPNB procedure itself. We assessed the quality of Peng et al [19] using Cochrane collaboration’s tool, shown in Fig 4. We found it used a wrong random sequence generation to classify subjects, that may result in a defined distribution outcomes to investigator and subjects. The results may also be affected by blinding methods. In general, it caused selection bias and detection bias as well as the decline in credibility.

HR was an established sensitive measure of response to nociceptive stimuli [19,20]. Previous studies comparing DPNB with EMLA have demonstrated significantly lower HR throughout circumcision in infants receiving DPNB [16,17]. Howard et al [17] reported mean differences in HR during lysis, Gomco clamping, and recovery between patients receiving EMLA (146.9±2.7 bpm) and DPNB (139.0±2.8 bpm). Butler-O’Hara et al [16] reported an increase in mean HR of 9 bpm from baseline throughout circumcision in infants receiving DPNB compared with 49 bpm for infants receiving EMLA. Lehr, V. T et al [15] also observed the largest mean difference in HR values between DPNB- and EMLA-treated infants at clamping (19±9.1 bpm) and cutting (18±9.6 bpm), although these were not significant (P = 0.13 and P = 0.18, respectively). Mujeeb Sabeen et al [21] demonstrated that the heart rate increased 12 times greater in EMLA group than the DPNB group. However, in this our study, there did not have any significant difference in analgesic efficacy between the study treatments according to the mean HR throughout the circumcision instead of several phase. Similar result was shown in Lehr, V. T et al [15] and Peng et al [19]. Measuring HR value at each phase instead of throughout the circumcision phases may have revealed significant differences in efficacy. It is great regret to gather nothing of HR at each phase from studies what we selected.

One interesting observation was that the higher mean NIPS scores in EMLA group compare to that of the DPNB group. Similarly, Benini et al [7] found attenuated pain response in infants receiving EMLA. In a study by Howard et al [17] EMLA was compared with DPNB. Distress score was significantly higher in infants treated with the EMLA. They concluded that DPNB with lidocaine was a more efficacious means of providing anesthesia for neonatal circumcision. Butler- O’Hara et al [16] found significantly lower scores on the NIPS, and Lander et al [4] found a significantly decreased crying time in the DPNB group compared with the EMLA group.

A higher incidence of hematoma was observed in the DPNB group. This finding was expected, due to the needle puncture at the base of the penis. No hematoma was observed in the EMLA group. There was no significant difference between the occurrence of edema and erythema in the EMLA and DPNB group. There are also some limitation in using of EMLA. The degree of absorption in EMLA cream cannot be predicted as it depends on many factors such as skin thickness and amount of ointment applied. There are no means of assuring uniform absorption of the EMLA cream given such factors as potential dilution by urine or differences in skin thickness in different neonatal populations. Application of the adhesive, semipermeable dressing that covers the topical cream may be problematic. In addition, monitoring is required to ensure that the dressing and cream remain in place. EMLA cream needs to be applied for at least one hour while lidocaine starts working in less than three minutes of application.

This is the first meta-analysis for comparison of anesthesia effect of DPNB and EMLA in circumcision. We use the randomized controlled trials with a higher level of evidence to perform this study, making the results more accurate and more reliable. However, there still some inherent limitations to our research. As with any meta-analysis, there was the unavoidable heterogeneity between different studies. Small study effects might also be a problem in these meta-analyses, which might lead to exaggerated summary estimates. However, the use of sensitivity analyses allowed us to explore the potential reasons for the observed heterogeneity. In addition, bias might exist for the published data in the other languages since our search was restricted to articles published in English and Chinese.

## Conclusion

The ideal method for intra-circumcision analgesia should have a frequent success rate with a small risk for complications. We hypothesized that if we could illuminate the effect of analgesia of EMLA cream and DPNB in providing pain relief, then more care will be given to patients. Our data showed that although DPNB may provide high risk of hematoma compared with EMLA, DPNB is a more effective method in pain relief in infants during circumcision. In the case of effectively reducing the side effect, according to this study, does DPNB more effective in reducing the patient's pain experience instead of EMLA.

## Supporting information

S1 File PRISMA 2009 checklist. (PDF)
